# Dynamic Causal Models and Physiological Inference: A Validation Study Using Isoflurane Anaesthesia in Rodents

**DOI:** 10.1371/journal.pone.0022790

**Published:** 2011-08-02

**Authors:** Rosalyn J. Moran, Fabienne Jung, Tetsuya Kumagai, Heike Endepols, Rudolf Graf, Raymond J. Dolan, Karl J. Friston, Klaas E. Stephan, Marc Tittgemeyer

**Affiliations:** 1 Wellcome Trust Centre for Neuroimaging, Institute of Neurology, University College London, London, United Kingdom; 2 Laboratory for Social and Neural Systems Research, Department of Economics, University of Zurich, Zurich, Switzerland; 3 Max Planck Institute for Neurological Research, Cologne, Germany; University of South Florida College of Medicine, United States of America

## Abstract

Generative models of neuroimaging and electrophysiological data present new opportunities for accessing hidden or latent brain states. Dynamic causal modeling (DCM) uses Bayesian model inversion and selection to infer the synaptic mechanisms underlying empirically observed brain responses. DCM for electrophysiological data, in particular, aims to estimate the relative strength of synaptic transmission at different cell types and via specific neurotransmitters. Here, we report a DCM validation study concerning inference on excitatory and inhibitory synaptic transmission, using different doses of a volatile anaesthetic agent (isoflurane) to parametrically modify excitatory and inhibitory synaptic processing while recording local field potentials (LFPs) from primary auditory cortex (A1) and the posterior auditory field (PAF) in the auditory belt region in rodents. We test whether DCM can infer, from the LFP measurements, the expected drug-induced changes in synaptic transmission mediated via fast ionotropic receptors; i.e., excitatory (glutamatergic) AMPA and inhibitory GABA_A_ receptors. Cross- and auto-spectra from the two regions were used to optimise three DCMs based on biologically plausible neural mass models and specific network architectures. Consistent with known extrinsic connectivity patterns in sensory hierarchies, we found that a model comprising forward connections from A1 to PAF and backward connections from PAF to A1 outperformed a model with forward connections from PAF to A1 and backward connections from A1 to PAF and a model with reciprocal lateral connections. The parameter estimates from the most plausible model indicated that the amplitude of fast glutamatergic excitatory postsynaptic potentials (EPSPs) and inhibitory postsynaptic potentials (IPSPs) behaved as predicted by previous neurophysiological studies. Specifically, with increasing levels of anaesthesia, glutamatergic EPSPs decreased linearly, whereas fast GABAergic IPSPs displayed a nonlinear (saturating) increase. The consistency of our model-based *in vivo* results with experimental *in vitro* results lends further validity to the capacity of DCM to infer on synaptic processes using macroscopic neurophysiological data.

## Introduction

Neural mass models have been used to simulate the electrophysiological response of cortical regions and have recently served as generative models for empirical M/EEG and LFP data [1], [2], [3], [4], [5], [6], [7], [8], [9]. These models furnish mathematical descriptions of detailed physiological processes including thalamic burst firing [1], spike frequency adaptation [10], neuronal noise [11], nonlinear channel conductances [12] and neuromodulation [13]. Of particular interest to empirical neuroscience is the inversion or fitting of these generative models to real experimental data, where mechanistic hypotheses regarding the genesis of data features can be tested. Dynamic causal modelling (DCM) provides a general framework in which neuronal ensemble models are inverted or ‘fitted’ to data. A particular ensemble model, known as an alpha-kernel model [14] is often used within DCMs of M/EEG and LFP data. The form of the dynamics is constrained by parameters that encode the strength of transmission at different types of synapses. Clearly, it is important to provide construct validity for these model parameters and ensure that they have a physiological interpretability. In this paper, we address this issue using LFP signals, acquired by invasive recordings in rat auditory cortex, under different levels of anaesthesia. This work is one from a series of ongoing validation studies of the models employed in DCM for electrophysiological data [15] using invasive recordings. Here, we focus on the ability of DCM to infer on specific aspects of synaptic transmission, i.e., whether it obtains plausible estimates of experimentally induced changes in transmission at excitatory glutamatergic synapses vs. inhibitory GABAergic synapses.

Pharmacological interventions can manipulate aspects of synaptic processing and can thus be used to validate model predictions: Here, we use isoflurane, a volatile anaesthetic agent that is used commonly in animal laboratory studies [16]. While, compared to other pharmacological agents, it induces a diverse range of molecular mechanisms leading to changes in synaptic signalling both pre- and postsynaptically, the resulting net effect at the neuronal circuit level is a decrease in excitation and an increase in inhibition [17]. Studies of specific presynaptic and postsynaptic effects of volatile anaesthetics have demonstrated actions on both the release of neurotransmitters and the function of neurotransmitter receptors [18]. Particular attention has been on inhibitory neurotransmission, where increased inhibition in the presence of isoflurane has been attributed to a sensitisation of GABA_A_ receptors [18], but also to increased synaptic release of GABA [19]. Glutamatergic neurotransmission has also been reported to be directly affected by isoflurane. Isoflurane reduces the strength of synaptic signalling following activation of both non-NMDA [20], [21] and in some cases to a greater [22] or equal [23] degree of NMDA receptors, as well as leading to its diminished release [21], [24]. Sophisticated biophysical models of anaesthesia have been developed to explain the theoretical properties they induce, such as phase transitions and hysteresis at transitions of consciousness [25], [26] and to examine observed side effects such as epilepsy [27], [28]. In this study, we use a coarser neural mass model [29] that embodies a lumped representation of biophysical processes underlying synaptic functions. In other words, processes such as presynaptic release and reuptake of transmitters or binding of transmitters to postsynaptic receptors are not modelled explicitly. Instead, the model absorbs these detailed processes into a slightly more abstract representation, modelling postsynaptic effects as the convolution of presynaptic inputs with postsynaptic kernels [4]. The magnitude of these synaptic kernels summarizes the strength of transmission at specific types of synapses. While less biophysically detailed than some previously proposed models mentioned above, this alpha-kernel model is currently most often used by experimentalists applying DCM to M/EEG data, e.g. [30]. We should emphasize, that the purpose of this paper is not to use a model for providing new insights into the mechanisms of isoflurane. Instead, we use isoflurane to induce known changes in the balance of excitatory and inhibitory transmission in order to test whether our model can infer these net changes correctly, given measured local field potentials. In the following, we describe our model in some more detail.

DCM is a generic modeling approach for inferring on the physiological mechanisms underlying measured neuroimaging data [31]. For MEG, EEG or LFP data, detailed biophysical neural mass models serve as generative models for both evoked, time domain data [4], [32] and steady-state, frequency domain data [29]. In DCM for steady-state responses (SSR) the auto and cross-spectra, for active regions or sources in the model, are predicted using their modulation transfer functions, augmented with white and *1/f* type spectral noise [10]. The model describes dynamic synaptic interactions among connected assemblies of different neuron types within brain regions (sources) as well as directed connections between brain regions. Each source is modelled as a layered macrocolumn comprising three interconnected cell populations with excitatory spiny stellate cells (assigned to granular layer IV), excitatory pyramidal cells and inhibitory interneurons (occupying both supra- and infra-granular layers; [2]). The dynamics are prescribed by two mathematical operators applied to the hidden neuronal states of each subpopulation. These are an input (synaptic) convolution kernel, which converts presynaptic firing to a postsynaptic membrane potential, and an output sigmoidal function that relates mean postsynaptic potential to an average firing rate [33]. Parameters of the model include maximum excitatory and inhibitory postsynaptic potentials and excitatory and inhibitory time constants, gain parameters describing ensemble firing efficiency and intrinsic connectivity that encode the efficacy of signalling among subpopulations within a source [10]. In addition, the signalling among sources is described with extrinsic coupling parameters. Crucially, these extrinsic connections can be of a forward, backward or lateral type, depending on the subpopulations targeted by afferents from the pyramidal population of each source [34]. Specifying different arrangements of forward and backward connections enables competing hierarchical architectures to be compared, using empirical data.

DCM for SSR assumes small perturbations about a dynamic equilibrium, where the perturbations are caused by endogenous fluctuations in cortical activity, i.e. white or coloured noise. The frequency response of a network of regions is described using the cross-spectral density of outputs, comprising auto- and cross-spectral components. Variational Bayesian techniques allow us to invert this generative model given real data and provide posterior densities over the parameters and the model evidence [35]. A Bayesian approach allows the model parameters to be constrained using physiologically plausible priors (c.f. Table 1 in [29]). In this validation study, the parameters we are particularly interested in comprise synaptic parameters encoding the amplitude of population responses to presynaptic glutamate release, from pyramidal and spiny stellate cells, and to GABA release by interneurons. Since the postsynaptic kernels encode mass action responses, their magnitude is a summary index of postsynaptic gain (determined by various biophysical properties such as receptor density and receptor “sensitivity”; e.g., conformational changes under isoflurane).

Previous validation studies of inference on synaptic processing using this DCM have used microdialysis measurements of extracellular glutamate levels to predict the parameter estimates that should be obtained by the model [15]. In this study, we apply a complementary test of both excitatory and inhibitory neurotransmission concomitantly, using different levels of isoflurane and a within-animal design. Under four levels of isoflurane 1.4%, 1.8%, 2.4% and 2.8%, we recorded local field potential measures from A1 and PAF under white noise stimulation and in silence, respectively, for twenty minutes. The spectral data from these time series formed the basis of our model inversion. Studies of isoflurane at similar doses in rats have reported a nonlinear (saturating) increase in GABAergic synaptic transmission with increasing isoflurane dose [19], [36], and a linear decrease in glutamatergic transmission [37]. We hoped to find that these dose effects would be reflected in our model parameter estimates.

## Materials and Methods

### Surgical Treatments

For recording LFPs in Lister hooded rats, a telemetric recording system (TSE Systems) was assembled, using chronically implanted epidural silverball electrodes above left and right auditory cortex in seven animals. In three of these animals, surgery and recordings were performed bilaterally and the results presented below use averages over both hemispheres.

Prior to surgery, rats were placed in an anesthesia box that was perfused with isoflurane (5%) mixed with 30% oxygen (O_2_) and 70% nitrous oxide (N_2_O). Once deeply anaesthetized, rats were transferred into a stereotactic frame and fixated using ear bars and a tooth bar. During surgery, animals inhaled a similar mixture of gases through a mask (isoflurane reduced to 2–3%). Body temperature was kept constant at 37°C using a heating pad feedback controlled by means of a rectal probe.

**Figure 1:**
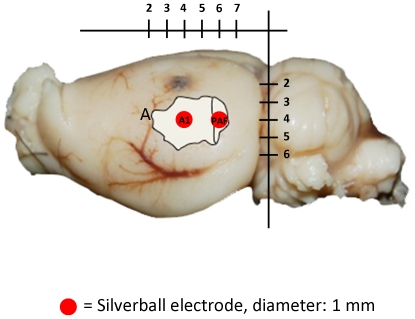
Electrode Placement. Electrode placement (silverball electrodes) in primary auditory cortex (A1) and posterior auditory field (PAF) in auditory cortex (A). The anatomical labelling of auditory fields was taken from [38] and matched to a rat brain from our animals. The indicated scaling is in mm.

Guided by stereotaxic coordinates, two electrodes were positioned above each hemisphere. When placing the electrodes, the temporalis muscle was partly removed and a cranial window was opened with a dental drill. Silverball electrodes were positioned epidurally above the primary auditory area, A1, (Figure 1) (4 mm posterior to bregma) and above the posterior auditory field, PAF (6 mm posterior to bregma) 7.6 mm lateral at a depth of 4 mm, targeting a primary and a non primary auditory cortex, respectively [38]. A fifth electrode, to which all recorded signals were referenced, was placed 5 mm anterior to bregma over the frontal sinus. The telemetry socket, to which electrodes were soldered, was fixed onto the head with dental cement.

All experimental procedures were approved by the State Agency for Nature, the Environment and Consumer Protection under file number 9.93.2.10.35.07.056, controlled by the veterinary authorities of the city of Cologne, and supervised by the Institute's animal protection officers.

### Pharmacological Interventions and Stimulus Conditions

At the beginning of each experiment, rats were placed in an isoflurane-perfused box for anaesthesia induction. Afterwards, animals were transferred into a sound shielded chamber and placed on the heating pad. During electrophysiological recordings, the heating pad was not turned on in order not to disturb the measurements. Temperature was verified between recording epochs and animals were warmed if necessary.

The experiment started with the lowest dose of isoflurane anaesthesia (1.4%) and was increased to the next level after 40 min. Each level of anaesthesia was accompanied by LFP recordings with 20 min continuous white noise stimulation, followed by 20 min under silence. White noise stimuli had a level of 83 dB (sampling rate 25 kHz) and were delivered by an RX6 processor and two free field magnetic speakers (Tucker Davis Technologies, TDT) that were placed with a distance of 15 cm, on both sides of the rats head. Recordings started immediately after increase of anaesthesia to the next level.

### Electrophysiological Recordings and Spectral Analysis

Electrode recordings were amplified (×1000) in the transmitter. Data were transferred to a receiver at a transmission frequency of 400 to 434 MHz, and amplified again (×10). Analogue LFP recordings were analyzed using a data acquisition system (DasyLab, Version 9.0, 2005, National Instruments) at a sampling rate of 2 kHz. Digital filtering was applied online (0.6–60 Hz).

The first ten minutes of each 20 min recording (under white noise and silence respectively) were extracted from the continuous time domain data and down-sampled to a sampling rate of 125 Hz. Frequency domain data-features were constructed from these epochs using a vector auto regression (VAR) model of order 8 (*p*). Channel data *y*, from the two channels (A1 and PAF) was modelled as a VAR process (using the SPM Spectral Toolbox: http://www.fil.ion.ucl.ac.uk, [39]).
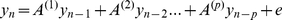
(1)


The autoregressive coefficients *A^(n)^* and channel noise covariance *E_ij_*, estimates were used to compute the cross-spectral densities for frequencies 1–30 Hz using the following transform:
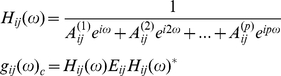
(2)


### Dynamic Causal Modelling and Bayesian Model Selection

**Figure 2:**
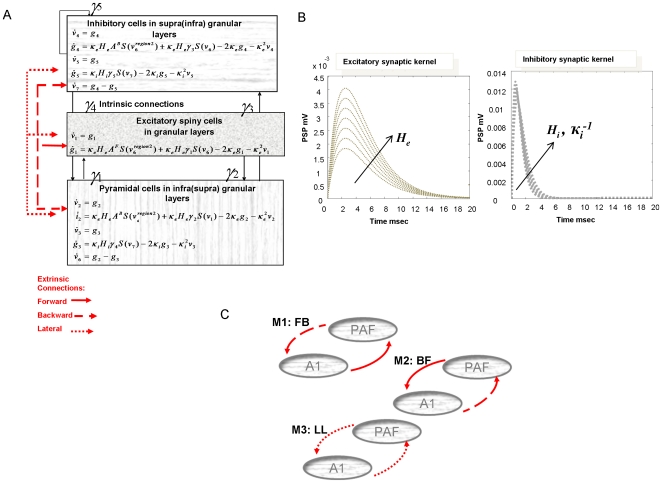
DCM and the Neural Mass Model. **A** Neural mass model used to represent regions in auditory cortex. Three cell subpopulations contribute to the ongoing dynamics. These include spiny stellate cells in granular layer IV, pyramidal cells and inhibitory interneurons in extra granular layers (II & III and V & VI). Intrinsic connections link dynamics between subpopulations in each source. Dynamic states include currents, *g*, and membrane potentials *v*. Extrinsic connections enter at specific cell layers. **B** Functions controlling ongoing dynamics and their parameterisation. Left: Excitatory synaptic kernel, which is convolved with the input firing to produce a depolarising change in membrane potential. The function is parameterised by its height *H_e_* and time constant. *H_e_* is allowed to mediate the effects of isoflurane. Increases in *H_e_* produce different responses, as per the arrow. Right: Inhibitory synaptic kernel, which is convolved with the input firing to produce a hyperpolarising change in membrane potential. The function is parameterised by its height *H_i_* and rate constant ***κ_i_***. Both can mediate the effect of isoflurane. Increases in these parameters produce different responses as per the arrow. **C** Three competing hypotheses regarding extrinsic connectivity in hierarchical auditory cortex, embodied by model 1, with forward connections from A1 to PAF and backward connections from PAF to A1 (M1:FB). The reverse architecture is constructed for model 2 (M2: BF). Model 3 contains lateral connections between the regions (M3: LL).

Dynamic causal models treat distributed brain networks as a connected set of neuronal ensembles or sources, where each ensemble (e.g., macrocolumn) is described by a set of differential equations. These equations
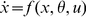
(3)describe the time evolution of states 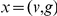, which are the membrane potentials 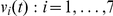 across and currents 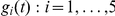 flowing through three cell populations in each macrocolumn and from which a frequency domain response can be computed (Figure 2A). The measured LFP is assumed to be dominated by the pyramidal cell membrane potential 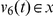 due to the parallel orientation of their apical dendrites [40]. Stellate cells and interneurons are assumed *a priori* to contribute less aggregate signal, comprising about 20% of the measured response [41]. These cell populations are modelled as layer specific; with spiny stellate cells in the granular layer reciprocally connected to pyramidal cells in infra- and supragranular layers. Inhibitory interneurons in the infra- and supragranular layers are in turn reciprocally connected to the pyramidal cells (Figure 2A). The dynamics are described by two functions describing synaptic and axonal output. The synaptic input-output function prescribes a convolution operator where presynaptic firing from one population is convolved with the postsynaptic response, either excitatory or inhibitory (Figure 2B) of another, mediated by intrinsic connections with strengths 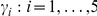 (Figure 2A). The second operator transforms membrane potentials to an output firing rate through a static sigmoid, 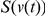.

The parameters *θ* encode synaptic inputs in terms of the amplitude of excitatory and inhibitory postsynaptic potentials (mE/IPSP; *H_e_* and *H_i_* in Figure 2B), rate constants (*κ_e/i_*) and the parameters of the static sigmoidal firing curve (*ρ_1,2_*). Forward, backward and lateral connections between regions (*A^F,B,L^*) originate and terminate at specific cell layers (Figure 2A). In this way DCM for SSR allows one to build hierarchical brain networks with connectivity rules as suggested by anatomical studies [42]. As in other sensory systems, the auditory system operates with connections that are largely bidirectional in nature and have laminar specificity depending on the hierarchical relation of the areas involved. All connections originate in pyramidal cells (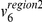 in Figure 2A). Forward connections terminate in granular layer IV [43], [44]. In contrast, reciprocal backward connections terminate primarily outside of layer IV [43], [45], [46] and lateral connections impinge on all cell layers. While our model lumps together supra- and infragranular pyramidal cells, it maintains the general asymmetries of connectional patterns in hierarchically related areas (Figure 2A).

We tested whether we could infer the known hierarchical relation of the primary auditory cortex (A1) to the posterior auditory field (PAF) in the auditory belt region, from the steady state LFP data. This hypothesis was tested using Bayesian model selection (BMS) based on the evidence for competing models [47]. Any given DCM represents a specific probabilistic mapping from experimentally controlled manipulations via neuronal dynamics to observed data. The goodness of this mapping (model) can be evaluated by the log model evidence (i.e., the log probability of observing the data given the model) which trades-off model accuracy and complexity in a principled way [39], [45], [47]. When comparing any two models, their log-evidence difference can be exponentiated to give the Bayes Factor (BF) which represents the ratio of the evidences. Conventionally, a Bayes factor *BF*>150 is considered very strong evidence in favour of one model over another (log Bayes Factor of ∼5). For larger systems, one could employ a network discovery approach [48], which would identify the sparsity structure in terms of which connection set in a fully connected Bayesian graph (model) best describe the data. However, here we deal with a very small (two-region) network, comprising A1 and PAF, with known reciprocal connectivity where our question was not *whether* connections played a role in generating the data but *what type* of connections generated the data. In model 1, we specified a two region network comprising A1 and PAF, where forward connections linked A1 to PAF and backward connections mediated the influences of PAF on A1, conforming to the hierarchical, forward and backward cortico-cortical connectivity structure of auditory cortex [49]. A second (null) hypothesis was instantiated by model 2, where hierarchical connectivity rules were inverted, with backward connections from A1 to PAF and forward connections from PAF to A1. Finally a third model with lateral connections was used to investigate whether recordings contained hierarchical asymmetry (Figure 2C).

### Bayesian Model Inversion and Parameter Estimates

The model was inverted (identified or fitted) by applying it to the cross-spectral densities from each of the ten hemispheres separately. In DCM, a variational Bayesian scheme is used, which factorises the conditional (posterior) density over unknown parameters into Gaussian marginal densities (here comprising model parameters and the log-precision of observation noise). Model inversion furnishes the (approximate) conditional density *q(θ)*, by maximising the negative free energy
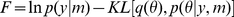
(4)where KL is the divergence between the true and approximate posterior. The negative free energy is hence a lower bound on the log model evidence, ln *p*(*y|m*). Note that the model evidence, also known as the marginal likelihood, evaluates the relative goodness of models by taking account of both the accuracy with which it can explain (fit) the empirical data features and the complexity of the model. The complexity term accounts for both the “effective degrees of freedom” (number of parameters and their interdependencies) and differences between the parameter estimate and its *a priori* value. Simply speaking, a model is more complex (i) the more parameters it has, (ii) the more independent (low covariances) and “flexible” (low precision) these parameters are, and (iii) the more the posterior is required to deviate from the prior to account for the data [35]. The free energy bound on log-evidence is used for model selection when testing a series of possible neural architectures. In this case the forward-backward scheme (model 1) was compared to the backward-forward scheme (model 2). A fixed effects analysis of the models was performed using the group Bayes factor [47], [50], with log-evidences averaged over hemispheres for those animals with dual recordings. The posterior densities from the best performing model are then used to provide the conditional mean and variance of our synaptic parameters of interest.

In our DCM, we modelled four conditions, corresponding to 1.4%, 1.8%, 2.4% and 2.8% isoflurane. The effect of isoflurane was modelled separately for each condition, allowing for unconstrained differences in specific synaptic parameters across increasing depths of anaesthesia. Three parameters were allowed to change across anaesthesia levels and thus explain condition specific effects on the spectral densities. These parameters were the maximum excitatory postsynaptic potential (EPSP), the maximum inhibitory postsynaptic potential (IPSP), and the inhibitory rate constants (*κ_i_*) of regions A1 and PAF. The quantitative effects of anaesthesia on these animal-specific parameters (averaged over hemispheres where applicable) for each region were entered into an ANOVA with anaesthetic depth as a factor. As we predicted that excitatory parameters decrease and inhibitory parameters increase with level of anaesthesia, we used one-tailed probabilities at *P*<0.05. To test for particular parametric effects, first and second order polynomials were later fitted to these animal-specific modulatory effects. We tested for consistent parametric effects from the polynomial coefficients using a one-tailed *t* test.

## Results

### Spectral Estimates

**Figure 3:**
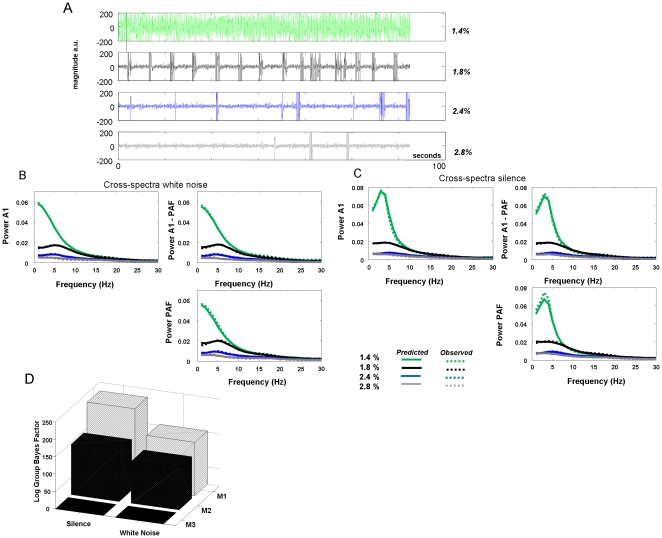
Modelled Data. **A** Time series recording from one animal in the noise condition showing increased burst suppression with increasing isoflurane dose. **B** Average cross-spectral density matrix representing spectral responses with prominent low frequency components for four isoflurane dose levels (Hashed line: 1.4%: green, 1.8%: black, 2.4%: blue, 2.8%: grey) as rats heard a white noise stimulus. Significant differences in spectral power are found for LFP recordings from A1 and PAF and also for their cross-spectra (off-diagonal term). Fits from model 1, averaged across animals are shown as full lines. **C** Average cross-spectra as per B, but for recordings and subsequent fits from the silent environment. **D** Log-evidence differences at the group level (relative to worse performing model M3: LL), showing very strong evidence in favour of model 1 (M1: FB) for both noisy and silent environments.

LFP recordings from A1 and PAF were collected after each anaesthetic administration for both white noise and silent auditory conditions. An examination of our time series data revealed burst activity at low doses of anaesthetic, which dissipated progressively with higher doses in line with the known burst suppression effects of isoflurane (Figure 3A) [51]. Cross spectral density measures were obtained from continuous ten minute epochs, comprising quasi steady-state representations. Across increasing dose levels, these spectra reflected the burst suppression as a decrease in low-frequency power [52], [53]. In other words, in the spectral domain, the features of our data reflected dose-dependent expression of bursts corresponding to low-frequency oscillations, whose power declined with increasing levels of anaesthesia. Altogether, our spectral measures reflect the statistical regularities of the data across time and provide a quasi steady state summary of the data across the measurement period.

We examined frequency differences (across levels of anaesthesia) within traditional EEG bands by binning spectral measures per animal (*n = 7*) (Figures 3B and 3C). Anaesthetic levels induced a difference in spectral power in primary auditory cortex for both white noise and silent conditions in the delta (1–4 Hz; noise: *p*<10^−8^, silence: *p*<10^−4^), theta (4–8 Hz; noise: *p*<10^−8^, silence: *p*<10^−4^), alpha (8–16 Hz; noise: *p*<10^−7^, silence: *p*<10^−6^) and beta (16–30 Hz; noise: *p*<10^−4^, silence: *p*<10^−3^) bands. Similarly, the PAF auto-spectra showed a significant effect of anaesthetic depth (delta; noise: *p*<10^−8^, silence: *p*<10^−5^, theta; noise: *p*<10^−10^, silence: *p*<10^−6^, alpha; noise: *p*<10^−6^, silence: *p*<10^−6^, beta; noise: *p*<10^−5^, silence: *p<*10^−4^). Finally, the cross-spectral densities comprising the off-diagonal components of Figures 3B and 3C were also profoundly affected by varying the depth of anaesthesia (delta; noise: *p*<10^−7^, silence: *p*<10^−4^, theta; noise: *p*<10^−10^, silence: *p<*10^−5^, alpha; noise: *p*<10^−7^, silence: *p*<10^−5^, beta; noise: *p*<10^−5^, silence: *p*<10^−4^).

### Model Comparison

These cross-spectra served as data features for model inversion (see Methods). We tested three models for each data set and averaged across hemispheres, where dual recordings had been obtained. Model 1 contained two sources representing A1 and PAF, with intrinsic dynamics as per Figure 2A with forward connections from A1 pyramidal cells to layer IV stellate cells in PAF (Figure 2C). The reciprocal backward connections coupled PAF pyramidal cells to A1 extra granular layers. In Model 2 the extrinsic connections were reversed, with forward connections from PAF to A1 and backward connections from A1 to PAF. Model 3 used reciprocal lateral connections with afferents from pyramidal cells targeting all layers (Figure 2C). Using the (approximate) log- evidence, we tested whether the data were better explained by model 1, which conformed to the normal connectivity rules in hierarchical sensory systems [42], or models 2 and 3, which would support higher to lower and equivalent hierarchical level signal exchange respectively. For this purpose, we computed the group log-evidence by simply adding the log-evidences 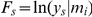 for each model 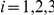 over subjects 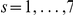. This assumes the data from each subject are conditionally independent. The resulting log odds ratio (log group Bayes factor; lnGBF) of model 1 relative to the second best performing model, model 2, revealed very strong evidence in favour of model 1 for both the white noise (lnGBF_12_ = 33.21) and silent (lnGBF_12_ = 80.63) conditions (Figure 3D). As can be seen in Figures 3B and 3C, the model fitted the spectral estimates from both conditions very accurately.

### Parameter Estimates of Glutamatergic and GABAergic Neurotransmission

Having established the most probable model, we next examined its parameters encoding glutamatergic and GABAergic neurotransmission. Defining 1.4% isoflurane as a baseline, we modelled condition-specific effects for trials at 1.8%, 2.4% and 2.8% isoflurane on parameters controlling the maximal amplitude of EPSPs and IPSPs and inhibitory rate constants *κ_i_* (see Methods). The maximum *a posteriori* (MAP) estimates (i.e., posterior means) for condition specific effects were used for statistical analysis at the group level. We first examined the overall changes in excitation and inhibition relative to baseline and observed significant decreases (*p*<0.005) and increases (*p<0.05*), respectively, in all stimulus conditions and in both auditory regions. Our analyses did not indicate any significant changes in inhibitory rate constants in A1 or PAF.

**Figure 4:**
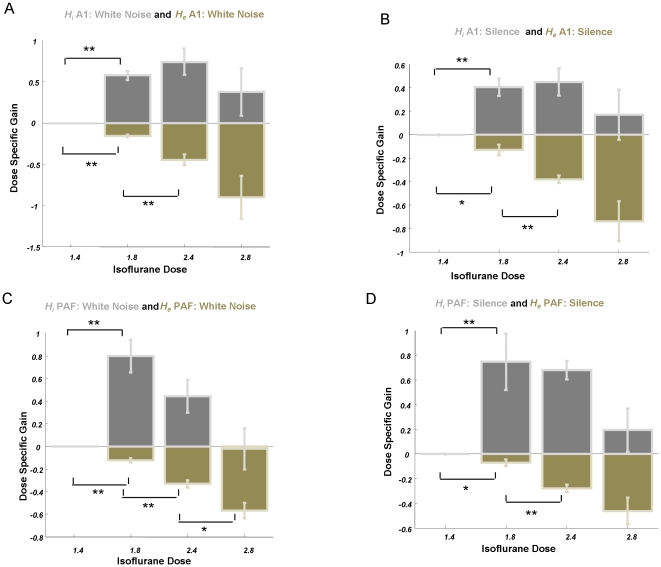
Parameter Estimates under Isoflurane. **A** Average dose responses at 1.4%, 1.8%, 2.4% and 2.8% for ***H_e_*** *(green)* and ***H_i_*** *(grey)* for region A1 from white noise condition (** p<0.005,* p<0.05; error bars denote s.e.m.). Overall trial effects are positive compared to zero baseline at 1.4% for the inhibitory parameters and negative for excitatory parameters. **B** Dose responses for ***H_e_*** and ***H_i_*** for region A1 from silence data. **C** Dose responses for ***H_e_*** and ***H_i_*** for region PAF from white noise data. **D** Dose responses for ***H_e_*** and ***H_i_*** for region PAF from silence data.

Dose-dependent analysis of synaptic parameters was performed using one-way ANOVAs for white noise and silent stimuli at A1 and PAF, with isoflurane depth as a single factor. In A1 there was a significant effect of isoflurane concentration on EPSP amplitude (noise: *F*_3,24_ = 6.11, *p* = 0.0031, silence: *F*_3,24_ = 9.26, *p* = 0.0003), Figures 4A and 4B. There was, however, only a trend towards differences in A1 inhibitory activity in terms of the postsynaptic amplitude (noise: *F*_3,24_ = 2.59, *p* = 0.076, silence: *F*_3,24_ = 1.88, *p* = 0.16 for both stimulus conditions) (Figures 4A and 4C). *Post hoc* analysis of primary auditory cortex revealed a significant decrease in EPSP amplitude for the 1.8% level compared to 1.4% (noise: *p* = 5×10^−6^, silence: *p* = 0.016, one-tailed *t*-test), a significant decrease for 2.4% compared to 1.8% (noise: *p* = 0.002, silence: *p* = 0.001, one-tailed *t*-test), and a trend towards a decrease for 2.8% compared to 2.4% (noise: *p* = 0.09, silence: *p* = 0.05, one-tailed *t*-test). The inhibitory trends were driven by significant increases for dose level differences at low doses (1.8%>1.4% noise: *p* = 1×10^−6^; silence: *p* = 0.0004, one-tailed t-test). Within the PAF (Figures 4C and 4D), both excitation and inhibition varied: ANOVA revealed a significant main effect of anaesthetic depth on EPSPs (noise: *F*_3,24_ = 28.89, *p* = 4×10^−8^, silence: *F*_3,24_ = 9.05, *p* = 0.0003) and on IPSPs (noise: *F*_3,24_ = 5.65, *p* = 0.0045, silence: *F*_3,24_ = 4.21, *p* = 0.016). *Post-hoc* we observed consecutive decreases in EPSP height in PAF similar to A1 (1.8>1.4; noise: *p* = 0.0001, silence: *p* = 0.027, 2.4: >1.8%; noise: *p* = 0.0002 silence: *p* = 0.0006; 2.8%>2.4%; noise: *p* = 0.01, silence: *p* = 0.09, one tailed *t*-test ), and this coincided with a significant increase in inhibitory neurotransmission as indexed by IPSP for 1.8% isoflurane compared to baseline (noise: *p* = 0.0003, silence: *p* = 0.009) that remained high for higher doses (2.4%>1.4%; noise: *p* = 0.001, silence: *p* = 3×10^−6^, 2.8%>1.4%: noise: n.s., silence: *p* = 0.19, one tailed *t*-test). Note that EPSP effects in A1 (silence and white noise), EPSP effects in PAF (silence and white noise) and IPSPs effects in PAF (white noise) survive Bonferroni correction for 8 multiple comparisons.

### Parametric Effects of Isoflurane: dose-response curves

**Figure 5:**
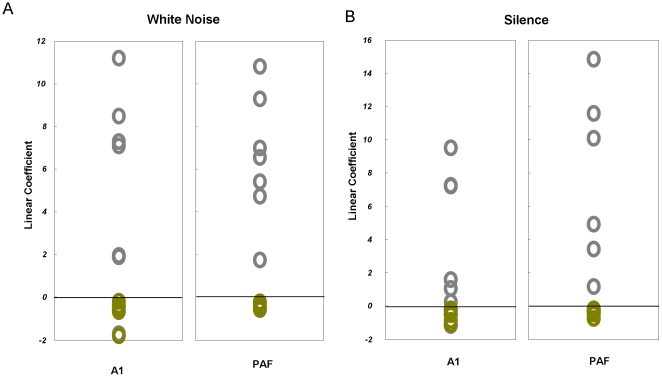
Dose Response Curves. **A** Linear components of polynomial fits for each animal individually in noise conditions for regions A1 and PAF, using a linear regression to describe the dose response of (conditional) EPSP effects (green) and using a second order function to describe the dose response of (conditional) IPSP effects (grey). **B** Linear components of polynomial fits for each animal individually during silence, for regions A1 and PAF obtained as per A.

To investigate the parametric effects of isoflurane depth, we estimated dose-response curves, using the parameter estimates above. We first used simple linear regressions to establish whether anaesthetic depth changes excitatory and inhibitory postsynaptic potentials in the expected direction. We then used a polynomial dose-response curve to assess the prediction that isoflurane produces a saturating nonlinear (decreasing) effect at higher doses (i.e., negative second-order term). A linear curve was fitted to the dose responses of EPSP and IPSP MAP estimates. In A1, we found a consistent linear effect for EPSP estimates, where slopes were negative in all animals for noisy and silent conditions (noise: −0.62±0.20; mean ± s.e.m., silence: −0.51±0.13); see Figure 5. Similarly, in PAF, isoflurane produced linear decreases in EPSP for doses from 1.4% to 2.8% in both environments (noise: −0.40±0.05, silence: −0.32±0.07). Animals showed variable positive and negative linear dose response curves for IPSP measures. However, a second order polynomial model revealed consistent effects across A1 and PAF for noise and silent stimulus in all but one case with negative second order effects and positive linear effects in both conditions (linear coefficient in A1; noise m = 5.18±1.96, silence: 4.20±1.59, and in PAF; noise: 6.51±1.12, silence: 5.57±2.58), Figure 5.

### Summary

In short, both inference on models and inference on the parameters of the model selected provide further endorsement of DCM as a way of accessing hidden architectures and synaptic physiology, given seemingly unresolved electrophysiological data. Our model comparison indentified the hierarchical architecture that was consistent with the known microanatomy of sensory brain systems, in terms of the laminar specificity of forward and backward connections. Furthermore, our pharmacological manipulation produced expected quantitative changes in hidden parameters encoding specific postsynaptic responses. We were able to generalise these parametric changes over two different contexts: the presence and absence of auditory noise in the environment. These two environments test the basic assumption in DCM for SSR that the cortical nodes can be understood as filters of surrounding cortical noise. In other words, even in silence there is sufficient cortical noise (white and pink components) to drive through the modulatory transfer function embodied by the neural state equations to produce the spectral output. The fact that no external perturbation was required to differentiate these models also points to a key difference between this DCM for steady state responses and models of evoked transients [5]. That is, while both DCMs are based on an input-state-output model, DCM for SSR uses the brain's own endogenous fluctuations as the input.

## Discussion

Using electrophysiological recordings of LFPs during different levels of isoflurane-induced anaesthesia, we have shown that by inverting a biologically plausible generative model of cortical dynamics one can recover latent quantities, such as the membrane responses to glutamate and GABA receptor binding. These conclusions were based on epidural recordings, which reflect, most prominently, membrane potential changes (net excitation and inhibition) of synchronous activity at pyramidal cell dendrites in an open field arrangement. Pyramidal cell apical dendrites are spatially aligned perpendicular to the cortical surface, producing a linear summation of currents [40], [41], while other cells such as spiny stellate neurons contribute less to the measured response, due to their closed field arrangement [41] where dendrites are oriented asymmetrically. By applying similar dipolar models that account for brain and tissue impedances, source localisation techniques can be applied to non-invasive EEG and MEG recordings to recover analogous focal cortical activity [54]. Though fundamentally ill-posed, plausible assumptions about the sources generating data makes this model inversion possible for both evoked and ongoing steady-state activity [55], [56]. Hence the methodology described and validated here serves as a motivation for similar non-invasive estimates of neurotransmitter-specific aspects of synaptic processing [15].

### Significance of this study for DCM analysis

DCM was originally designed for the analysis of fMRI time series [56] to uncover the strength of directed connections between brain regions activated by experimental perturbation. More sophisticated neural state equations were used in subsequent DCM implementations for non-invasive electrophysiological data (M/EEG) [4], [5] and invasive (LFP) recordings [29]. Several validation studies have shown been performed previously [31]. For example, simultaneous electrophysiological recordings and fMRI showed that DCM for fMRI could infer the origin of epileptic activation spread [57]. Other work demonstrated that DCM for SSR could detect known changes in synaptic transmission following a developmental perturbation of extracellular glutamate levels [15]. The validation presented here goes further on two levels. First we have validated the ability of DCM for SSR to distinguish between excitatory (glutamatergic) vs. inhibitory (GABAergic) synaptic transmission in cortico-cortical connections. Secondly, we examine a dose response, showing that DCM can distinguish between different degrees of drug-induced synaptic effects. For this, we examined a hierarchical sensory structure using invasive recordings and inferred a connectivity architecture that is predicted by anatomical data [42].

### Evidence for Architectural and Dose Response Inference

Using electrophysiological recordings, we found that a model with forward, driving connections from primary auditory cortex to the posterior auditory field and backward connections in the opposite direction outperformed a model connected reversely and a model with lateral connections specifying two regions at the same hierarchical level. The (first) anatomically plausible model was inferred with strong evidence for both silent and noisy environments.

The present view of isoflurane action is that it affects both excitatory and inhibitory synaptic transmission, influencing pre- as well as postsynaptic processes; the net effect is a decrease in excitation and an increase in inhibition [17]. Concerning inhibitory neurotransmission, increased inhibition due to isoflurane has been attributed to a sensitisation of GABA_A_ receptors [18] and increased presynaptic release of GABA [19]. Notably, the increase in inhibition with higher isoflurane levels has been found to show a nonlinear (saturating) form [19]. In some studies of spontaneous IPSPs, evidence of a paradoxical reduction of IPSP amplitude has been observed [58], however when including both evoked and spontaneous IPSPs, findings show a net increase in the transfer of negative charge to the postsynaptic cells [59]. Our model makes no distinction between these two types of post-synaptic responses and so we consider the parameter estimates in terms of the drug's net effects. Moreover pre- and post- synaptic measures found empirically (for example IPSP amplitude and frequency) should be included in the expected net effect, and indicate an overall increase in inhibition. With regard to excitatory neurotransmission, isoflurane diminishes glutamate signalling [20], [21], probably due to diminished presynaptic release of glutamate [21], [24]. Specifically, at concentrations similar to those used here, near linear depression of presynaptic glutamate release has been found [17], [37].

It was reassuring to see that our model inversion results were consistent with these empirical findings, showing a linear decrease in peak, or maximum EPSP amplitudes with increasing isoflurane concentration. Moreover, our model parameter estimates also showed the expected changes in inhibitory synaptic processes with isoflurane depth, with nonlinear increases in GABAergic neurotransmission beyond the 1.4% baseline (Figure 4).

### Current Limitations

Our model is limited by the receptor characteristics that determine its dynamic repertoire. The neural mass model employed here employs fast linear postsynaptic ion channels, both at excitatory and inhibitory synapses. Other receptor types not included in our model, such as glutamatergic NMDA and cholinergic receptors, have also been shown to be affected by isoflurane [22], [60]. Furthermore the parameters encoding EPSP and IPSP amplitudes represent lumped coupling parameters that quantify the collective effect of a number of biophysical processes such as receptor binding and transmitter reuptake. These are not separately amenable to the current model assay. Notwithstanding these limitations, our present investigations complement previous validation work [15] in demonstrating that DCM can be used to infer synaptic processes from mass, population measures of membrane potential fluctuations.

### Possible Application and Future Directions

DCM works on the principle that model parameters identifiably contribute to the dynamic processes controlling measurable brain responses. In the case of DCM for steady state and evoked responses, the dynamic processes are described by a neural mass model and detail how excitatory and inhibitory cells within a given region interact but also how signals are passed between the regions themselves. In this work, we have validated the inference that is made on these unobservable hidden states. Future work will involve the validation of similar synaptic assays using non-invasive measures including MEG or EEG where manipulations are similarly performed using pharmacological agents with known synaptic consequences. Further work will also examine the validity of synaptic assays from more complex neural models, e.g. those including non-linear NMDA channels [61]. Establishing such a framework would offer great potential to neuroscientists and clinicians interested in examining normal and pathophysiological synaptic processing in humans at a combined behavioural, brain network and synaptic level. This may be of particular relevance for establishing physiologically interpretable assays of synaptic function which hold promise for diagnostic categorisation of patients in psychiatric spectrum disorders, such as schizophrenia [62]. The approach may also help to elucidate synaptic effects induced by novel drug compounds.
